# Highly-efficient extraction of entangled photons from quantum dots using a broadband optical antenna

**DOI:** 10.1038/s41467-018-05456-2

**Published:** 2018-07-31

**Authors:** Yan Chen, Michael Zopf, Robert Keil, Fei Ding, Oliver G. Schmidt

**Affiliations:** 1 Institute for Integrative Nanosciences, IFW Dresden, Helmholtzstrasse 20, 01069 Dresden, Germany; 2 Institut für Festkörperphysik, Leibniz Universität Hannover, Appelstrasse 2, 30167 Hannover, Germany; 3 Chemnitz University of Technology, Reichenhainer Strasse 70, 09107 Chemnitz, Germany

## Abstract

Many quantum photonic technologies require the efficient generation of entangled pairs of photons, but to date there have been few ways to produce them reliably. Sources based on parametric down conversion operate at very low efficiency per pulse due to the probabilistic generation process. Semiconductor quantum dots can emit single pairs of entangled photons deterministically but they fall short due to the extremely low-extraction efficiency. Strategies for extracting single photons from quantum dots, such as embedding them in narrowband optical cavities, are difficult to translate to entangled photons. Here, we build a broadband optical antenna with an extraction efficiency of 65% ± 4% and demonstrate a highly-efficient entangled-photon source by collecting strongly entangled photons (fidelity of 0.9) at a pair efficiency of 0.372 ± 0.002 per pulse. The high brightness achieved by our source represents a step forward in the development of optical quantum technologies.

## Abstract (web summary)

Cascade radiative decay in quantum dots is a promising way of generating entangled photon pairs, but the extraction efficiency is a strongly limiting factor. Here, the authors demonstrate high extraction efficiency through a broadband dielectric photonic antenna.

## Introduction

Sources of entangled photons lie at the heart of photonic quantum information processing^1^. Since 1980s it is known how to generate pairs of entangled photons through the process of spontaneous parametric down conversion (SPDC), where a beam of photons is split into entangled pairs by passing it through a crystal^2,3^. Unfortunately, there is an intrinsic disadvantage of these sources. To avoid too much noise (i.e., multiple photon-pair events), the photon pairs can only be produced at a low per-pulse emission probability (*p*)^4^, typically *p* < 0.1 per excitation pulse. This inefficiency has thus been a major bottleneck^5,6^.

Semiconductor quantum dots (QDs) are a promising alternative as they are more efficient at producing entangled photon pairs^7^. One pair of entangled photons can be produced on demand with almost 100% probability through the radiative cascade decay from the biexciton (XX) state to the ground state via intermediate exciton states (X)^8^. Yet QD sources face their own set of challenges. The community has made a lot of progress recently in solving several key problems. A well-known one is that the small energy difference between the intermediate states, or fine structure splitting (FSS)^9^ caused by anisotropy of QDs, leaks “which path” information and reduces the quality of the entanglement. The final two-photon state varies in time, as $|\varphi\rangle = |H_XH_{{\mathrm{XX}}}\rangle + e^{{\mathrm{is}}\tau _X\hbar ^{ - 1}}\left| {V_XV_{{\mathrm{XX}}}}\rangle \right.$, where H represents horizontal polarization; V denotes vertical polarization; *τ*_X_ is the delay of exciton recombination and *s* is FSS^10^. To obtain a high entanglement fidelity (*F*) in time-integrated measurements, one needs to cherry-pick a QD with very small FSS $\left( {s < \hbar \tau _X^{ - 1}} \right)$. Recently, GaAs/AlGaAs QDs fabricated by nanohole etching and infilling have been made with small FSS energies and very short exciton lifetimes (typically <200 ps). They can emit transform-limited photons^11^. Highly indistinguishable and strongly entangled photons are also reported^12,13^. In the meantime progress has been made in tackling other challenges, such as the mediocre entanglement fidelity *F* at zero FSS^10^, and the color mismatching between dissimilar QDs^14^.

The faintness of QD sources remains a major hurdle. QDs are embedded in semiconductor material that has a high refractive index. Less than 1% of the photons escape; all others are back-reflected into the substrate. This means that entangled photon pairs can only be collected from QDs at an extremely low per-pulse emission probability (*p*~10^−5^ to 10^−4^ for typical samples), much lower than SPDC sources and too low to perform multiphoton experiments. The brightest SPDC sources have catalyzed recent breakthroughs such as demonstrating a ten-photon Greenberger–Horne–Zeilinger state^5,15^ and a satellite-based entanglement distribution^16^. It would not be feasible to do that with a QD-based source due to its limited performance.

Enhancing the efficiency by which photons can be extracted from QDs is therefore a priority. One way to do it is to fabricate a photonic structure around the QD^17–20^. Coupling QDs, both spectrally and spatially, with the cavity mode of the structure boosts and redirects the light. While this has been done for single photons, it is much harder to implement for an entangled photon source, as it requires the simultaneous Purcell enhancements of two photons with different energies. In a landmark experiment Dousse et al.^21^ realized a bright QD source, with a pair emission probability per pulse *p* of 0.12 and entanglement fidelity *F* of 67% by using a double-micropillar structure. The X and XX emissions were tuned into resonances with the two narrowband cavity modes. Although elegant conceptually, the double-micropillar structure is technologically complex and thus difficult to apply widely.

Encouraged by rapid advances in optical antennas, several groups are exploring extracting photons over a range of wavelengths from single quantum emitters. In plasmonic antennas (e.g., metallic slits, monopole or Yagi-Uda), emissions from luminescent molecules or QDs can be modified by placing them close (within a few tens of nanometers) to metallic nanostructures generating strong local fields^22–24^. However plasmonic antennas are lossy at optical frequencies and often only work with certain polarizations.

A promising alternative is to use broadband antennas made from dielectric or semiconductor materials^25–29^. For semiconductor QDs, great progress has been made with photonic nanowire or nano-trumpet antennas to achieve a photon pair efficiency *p* of ~0.0025 (with *F* = 81.7%)^30,31^. Micro-lenses with distributed Bragg reflectors (DBRs) as backside mirror have also been explored for extracting photons from QDs. To date they have delivered a single-photon extraction efficiency as high as 23%^32^. A further improvement is challenging as the reflection of the DBR mirror decreases strongly for incidence angles larger than 20° and a large portion of photons cannot escape. The nanometer QD positioning accuracy and precise lens fabrication are technologically demanding.

There are theoretical efforts as well. Ma et al.^28^ proposed to embed QDs in a metal-DBR cavity where the interference effects alter the internal angular power distribution, similar to the idea used in resonant cavity light emitting diodes. In this way a photon extraction efficiency of ~50% was calculated. Chen et al.^25^ suggested sandwiching a QD containing membrane between low refractive index layers to reduce the total effective refractive index. The main challenge to obtain the calculated efficiency of 99% using conventional lens systems (low NA) is to fabricate thin (~100 nm or less) QD membranes with high optical qualities^33^. Despite the urgent demand, an efficient entangled-photon source remains elusive.

In this work, we design and fabricate a QD-based entangled photon source of high brightness. It employs a broadband dielectric photonic antenna to beam polarization-entangled photons emitted from single QDs. We achieve a single-photon extraction efficiency of 65% ± 4%, for both X and XX photons. Under 76 MHz pulsed resonant two-photon pumping, ~28.3 MHz photon pairs are collected from single QDs with a high entanglement fidelity *F* of 90% ± 3% (without any temporal selection) and a single-photon purity of 99.8% (uncorrected). The photon pair efficiency *p* reaches a value of 0.372 ± 0.002, four orders of magnitude larger than that of a bare QD sample. The continuous wave two-photon pumping scheme is also discussed, which opens the door to a Gigahertz rate entangled photon source. This device is straightforward to make and robust, paving the way for a host of quantum photonics applications.

## Results

### Optical antenna design and fabrication

**Fig. 1:**
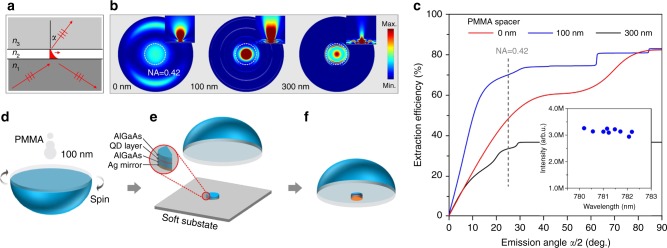
Dielectric antenna design and numerical results. **a** Illustration of light propagating at the media interfaces. The three media fulfill ${{n}}_1 \ge {{n}}_3 > {{n}}_2$ as in our design. The evanescent wave caused by total internal reflection at the interface will be coupled to the propagating wave when the intermediate gap decreases. **b** Far field view and cross section (inset) of dielectric antennas’ emission. PMMA spacers with different thicknesses, thus different coupling strength, are considered. The collection angle of a NA = 0.42 lens is indicated by the dashed circle. Antenna with 100 nm PMMA spacer gives the best result in terms of coupling strength and emission angle. **c**–**e** Fabrication flow of the device. The dielectric antenna consists of an AlGaAs membrane (with embedded QDs), a low refractive index PMMA spacer and the GaP solid immersion lens (SIL), with the refractive indices of *n*_1_, *n*_2_, and *n*_3_, respectively. A bottom silver mirror is used to reflect downward-emitted photons. **f** Numerical results for the dielectric antenna as a function of emission angle *α*. The collection angle of a NA = 0.42 lens is indicated by the dashed line. Inset shows the broadband operation of the dielectric antenna. All QDs randomly chosen across the membrane are bright

The GaAs QDs are fabricated by solid-source molecular beam epitaxy. In-situ Al droplet etching creates symmetric nano-holes on an AlGaAs surface that are then filled with GaAs and capped with AlGaAs. Statistics show that these QDs feature an average FSS of only (4.8 ± 2.4 μeV) and narrow wavelength distributions at the Rb D2 transitions^12^. The sample fabrication flow and its morphology are shown in Supplementary Figure 1. Details on the sample fabrication and its structure are described in Supplementary Table 1. The high refractive coefficient of AlGaAs (*n*_1_, 3.5) presents a great challenge to photon extraction efficiency. For a bare QD sample, virtually no light is coupled into propagating waves in free space beyond the critical angle of total internal reflection $\alpha _{c12} = {\mathrm{arcsin}}(n_2/n_1)$, where *n*_2_ is the refractive coefficient of free space. Thus only a tiny fraction of photons can escape from the QD host material. Instead, an evanescent wave is formed at the semiconductor/air interface, decaying exponentially in the normal direction. We propose that, by bringing a high refractive index material (medium *n*_3_, 3.4) close to the interface and accurately engineering the gap between them, the evanescent wave transforms into a propagating wave (Fig. 1a) and the photons traveling in directions beyond the critical angle *α*_*c*12_ are funneled efficiently into medium *n*_3_.

We employ a commercially available Gallium Phosphide (GaP) semi-spherical lens as the medium *n*_3_. Its refractive index *n*_3_ is very close to *n*_1_. To exclude Fresnel reflection at the GaP-vacuum interface, we deposit a layer of alumina (wit a thickness fulfilling the impedance matching condition) on top as an anti-reflection coating using atomic layer deposition. The GaP lens used in our experiment is large in diameter (2 millimeter). We select Polymethyl methacrylate (PMMA) with *n*_2_ = 1.6 as the intermediate layer and its thickness can be well controlled by adjusting the spin coating speed. A single QD membrane, which is prepared via wet chemical etching (Supplementary Figure 2a, b) with a reflecting silver layer, is glued to the center of the PMMA covered GaP lens. The QD layer is precisely positioned at the anti-node of the thin membrane cavity (see Supplementary Figure 2c). More details are given in Supplementary Note 2.

In the following we discuss the importance of our design. We first consider theoretically how the far field emission profiles and the photon extraction efficiency vary with the gap size, and the results are plotted in Fig. 1b, c. In the absence of a gap, as considered by Gschrey et al.^28^, Ma et al.^32^ and others, photons couple strongly into the lens but at the expense of a wide emission angle (*α*/2). For example, a theoretical extraction efficiency of 70% would require an objective with NA = 0.9, which has never been realized in practice^32^. A large gap of 300 nm leads to convergent emission but the extraction efficiency drops rapidly to about 30%. Our simulation shows that convergent emission and high extraction efficiency can be achieved simultaneously only for a small range of PMMA thicknesses (Supplementary Note 3). For 100 nm PMMA used in this work, a theoretical extraction efficiency of 70% is conveniently achieved with a NA = 0.42 objective. As comparison, we stress that without the lens, most of the photons are confined within the GaAs substrate (Supplementary Figure 3).

In typical proposals using solid immersion lenses (SILs), uneven/rough surfaces cause a large air gap between SIL and substrate surface, leading to a dramatic degradation of the extraction efficiency. To avoid this, we stick the QD-containing membrane to the lens, as the thin/flexible membrane smoothly adapts to the ‘wavy’ bottom surface of the GaP lens. The fabrication flow of the dielectric antenna is sketched in Fig. 1d–f. The refractive indices of corresponding layers can be found in Supplementary Table 2. More details are given in Supplementary Note 2.

Another important feature of this optical antenna is the broadband operation. In order to beam the energetically different X and XX photons simultaneously, the dielectric antenna needs to tolerate a broad wavelength range. The inset of Fig. 1e shows the measured count rate for nine randomly selected QDs inside the circular membrane (70 μm in diameter). All of them are equally bright, despite their different wavelengths and spatial locations.

### Device characterization

We first measure the QD photoluminescence (PL) in a helium flow cryostat system (Supplementary Figure 6). To characterize the collection efficiency, QDs are excited above-band by a pulsed laser at a repetition rate of 76 MHz. Strong X emission saturates the silicon charge-coupled detector (CCD) within a very short integration time (<0.1 s). For the randomly positioned QDs in the antenna (see Supplementary Note 4), we measure a single-photon flux of over 3 million counts per second on the CCD for most of the QDs and no deterministic nanoscale positioning of the QDs is required^32,34,35^.

**Fig. 2:**
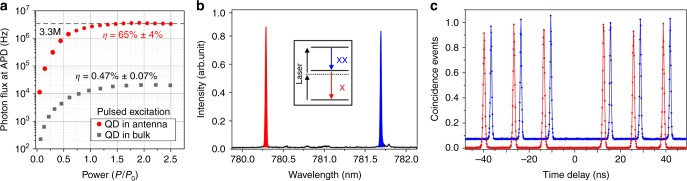
Characterization of the broadband dielectric antenna and resonant excitation of XX state under 76 MHz pulsed excitation. **a** Single photon flux measured on APD, plotted as a function of laser excitation power (in saturation units), for a QD in dielectric antenna (red dot symbol) and in unprocessed bulk substrate (gray square symbol). At saturation, 3.3 million of single photons per second are detected. **b** Resonant excitation of the XX state using a two-photon pump scheme. The residual laser signal is strongly suppressed by the setup. **c** Intensity-correlation histogram from the X transition under two photon pumping excitation. Nearly ideal single-photon emissions is demonstrated by the vanishing multi-photon events at zero time delay ${\boldsymbol{g}}^2(0) \cong 0.002$ for both X and XX photons

Power-dependent PL intensity of a QD in the antenna, together with that of a QD in the bare substrate, are recorded by an avalanche photodiode (APD). An enhancement of a factor >100 is clearly observed as is shown in Supplementary Figure 5. The count rate on the APD reaches 3.3 × 10^6^ at saturation (still, under 76 MHz pulsed excitation, see Fig. 2a). Taking into account the experimental setup efficiency (~6.7%, see Supplementary Note 4), the collection efficiency (single-photon extraction efficiency *η*) of the antenna at the first collection lens is 65% ± 4%. The unprocessed QD in bulk shows a collection efficiency of 0.47% ± 0.07% at saturation. Unlike the narrowband cavity-assisted sources, our antenna operates at a broad spectral range (Supplementary Figure 4). The high extraction efficiency applies to both X and XX photons.

### Resonant two-photon excitation

To generate polarization entangled photons from single QDs, the XX state must be effectively populated. Three pumping schemes—two-photon pumping^36,37^, phonon-assisted two-photon pumping^38–40^ and rapid adiabatic passage using chirped pulses^41^—could populate the XX state with high fidelity. We apply the two-photon pumping scheme here. A strong pulsed laser, at a frequency in between the X and XX transitions, is used to coherently excite the XX state through the absorption of two laser photons. Residual laser background noise is minimized by performing the experiment in a fiber-based closed-cycle cryostat system with a confocal setup and notch filters inserted in the beam. The setup is sketched in Supplementary Figure 6.

At the π pulse excitation, we obtain very pure, almost background-free emissions for both X and XX photons (Fig. 2b). The XX population fidelity under the two-photon pumping scheme is estimated to be 88%. More details are given in Supplementary Note 4. Auto-correlation measurements are performed and the raw data are presented in Fig. 2c. The absence of coincidence counts at zero time delay proves ultra-high purity single photon emission. The raw correlation function is measured to be $g_{\mathrm X}^2\left( 0 \right) = 0.002 \pm 0.002$ and $g_{{\mathrm{XX}}}^2\left( 0 \right) = 0.002 \pm 0.002$, respectively. The single-photon purities, which are defined by $[1 - g_{{\mathrm{XX}}}^2(0)]$ and $[1 - g_{\mathrm{X}}^2(0)]$, are therefore over 99.8% (without any background corrections). Both X and XX emissions show fast exponential decay, with lifetimes of 103 ps for the XX and 195 ps for the X (see Supplementary Figure 8 in Supplementary Note 6). The equal intensity of the X and XX emissions suggests that the two-photon pumping scheme is highly efficient.

The collected entangled photon pair efficiency is given by $r = r_{\mathrm{p}}\eta _{\mathrm{p}}\eta ^2[1 - g_{\mathrm{XX}}^2(0)]^{1/2}[1 - g_{\mathrm{X}}^2(0)]^{1/2}$ where *η*_p_ is the XX population fidelity, at an excitation repetition rate *r*_p_ of 76 MHz^25^. After correcting the XX population fidelity, we can collect ~28.3 MHz entangled photon pairs in the first lens, corresponding to a photon pair efficiency per pulse *p*~0.372 (0.002). It is worth mentioning that although the emission pattern of our antenna is highly directional, it does not have a perfect Gaussian distribution. For optical fiber-based applications, a further optimization of the current design may be required.

Thanks to the high excitation/extraction efficiencies, we are able to resonantly excite the QD under continuous wave two-photon pumping (see Supplementary Figure 7 in Supplementary Note 5). Considering the short lifetime of X and XX, the photon pair rate can, in principle, enter the Gigahertz range.

### Entanglement fidelity evaluation

Since the GaP lens is quite large, the dielectric antenna is insensitive to the lateral distribution of QDs. This is a major advantage over other photonic structures where the tolerances of fabrication and emitter displacement are small. There are plenty of QDs within one single device, thus leaving us more measurement freedom. A QD with a FSS of $s = 1.5 \pm 0.4\;{\mathrm{\mu eV}}$ is chosen in the experiment. XX and X photons are sent to a 50:50 beam splitter. Each output arm contains a quarter-wave plate, a half-wave plate and a polarizer for arbitrary basis projection. Then the photons are dispersed by a spectrometer to spectrally select XX and X photons at the first and second arm, respectively. Finally they are sent to single photon detectors.

**Fig. 3:**
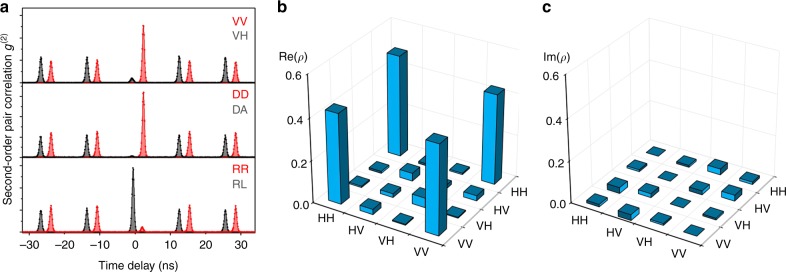
Entanglement fidelity of a quantum dot with finite FSS. **a** XX-X cross-correlation measurements under resonant two-photon excitation for co- and cross-polarized photons in the rectilinear (H horizontal, V vertical), diagonal (D diagonal, A anti-diagonal) and circular (R right-handed, L left-handed) polarization bases. The graphs for co-polarized (red) and cross-polarized (black) photons are shifted by 3 ns for clarity. A fidelity *F* = 90% is extracted. **b**, **c** Real (**b**) and imaginary (**c**) parts of the two-photon density matrix as reconstructed from 16 correlation measurements of the same QD by employing the maximum likelihood technique. The fidelity extracted from this matrix is *F* = 89%

Due to the high photon pair flux, very smooth and clean correlation data are obtained as depicted in Fig. 3a. Six cross-correlation measurements in the rectilinear (HV), diagonal (DA) and circular (RL) polarization basis are performed. Strong bunching in VV, DD, RL together with strong anti-bunching in VH, DA, RR show that the XX and X photons are entangled. The degree of correlation in one polarization basis is given by^42^

1 $$C_{{\mathrm{basis}}} = \frac{{g_{{\mathrm{XX,X}}}^2\left( 0 \right) - g_{{\mathrm{XX,}}\bar{\mathrm X}}^2(0)}}{{g_{{\mathrm{XX,X}}}^2\left( 0 \right) + g_{{\mathrm{XX,}}\bar{\mathrm X}}^2(0)}}$$

where $g_{{\mathrm{XX,X}}}^2(0)$ and $g_{{\mathrm{XX,}}\bar{\mathrm X}}^2(0)$ are the coincidences of co-polarized and cross-polarized bases at zero time delay. From the measurement data, the degree of correlation of three different basis sets are extracted:

2 $$C_{{\mathrm{linear}}} = 0.83 \pm 0.03$$

3 $$C_{{\mathrm{diagonal}}} = 0.93 \pm 0.03$$

4 $$C_{{\mathrm{circular}}} = - 0.84 \pm 0.03$$

The fidelity to the maximally entangled state is therefore obtained as:

5 $$F = \frac{{1 + C_{\mathrm{linear}} + C_{\mathrm{diagonal}} - C_{\mathrm{circular}}}}{4} = 0.90 \pm 0.03$$

Quantum-state tomography determines more precisely the quantum state of the photons^43^. To reconstruct the density matrix, 16 cross-correlation measurements with different base combinations are performed. The raw data are shown in Supplementary Figure 9. The maximum likelihood estimation is employed to find out the appropriate density matrix which is the closest to the measured results. The resulting matrix is shown in Figs 3b, c. From the density matrix, we extract a fidelity *F* = 89%, which is very close to the value obtained from formula (5). More details can be found in Supplementary Note 7.

### Comparison with state-of-the-art

**Fig. 4:**
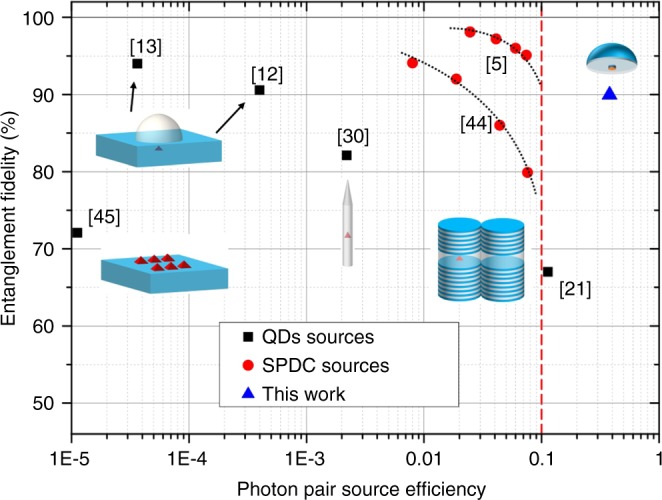
Comparison with other QD and SPDC entangled photon sources. State-of-the-art results, in terms of entanglement fidelity and source efficiency, on several different types of QD sources are shown as black symbols. High quality SPDC entangled photon sources are shown as red symbols, see also the work by Jöns et al.^30^. Red dashed line indicates the efficiency limit of SPDC sources. The performance of the current device is shown as a blue triangle in the upper right corner, marking the highest combination of source brightness and entanglement fidelity

A comparison with other QDs and SPDC sources is shown in Fig. 4. We plot the entanglement fidelity to a maximally entangled state as a function of the photon-pair source efficiency. Here the photon-pair source efficiency is defined as the probability of collecting a photon pair per excitation pulse in the first collection optics, the photon pair efficiency *p*. An ideal source would sit in the upper right corner of the diagram. Data for SPDC sources are presented as red circle symbols and data for QD sources as dark square symbols. A detailed description of how each individual data point was derived is discussed in a previous study^30^.

Because of the probabilistic nature of the photon pair generation in SPDC sources, these sources show Poissonian photon counting statistics. The source efficiency must be kept below the limit of 0.1 (represented by red dashed line) to suppress noise, i.e., multi-pair emissions. When the source efficiency is pushed towards 0.1, the entanglement fidelity degrades quickly, as illustrated by the gray dashed curve^5,44^. By contrast, the QDs can reach near-unity source efficiency without negatively influencing the entanglement fidelity. However, most QD sources to date are operated at extremely low efficiency (*p*~10^−5^ to 10^−4^)^12,13,45^. Dousse et al.^21^ have reported a record-high source efficiency of 0.12. Unfortunately the entanglement fidelity was low and the single-photon purity was low $( g_{\mathrm{XX}}^2(0) \ge 0.45)$, possibly due to the large FSS and the cavity mode feeding^46^. Moreover, the double-pillar device is very hard to make.

By comparison, our experiment (the blue triangle in Fig. 4) demonstrates a simple and robust optical antenna that achieves an unprecedented entangled photon pair efficiency *p* of $0.372 \pm 0.002$. This device achieves a combination of brightness, single-photon purity (99.8%) and entanglement fidelity (90%).

## Discussion

SPDC sources have been used as the main working horse in the quantum optics field for decades, but improving the brightness of these sources further is impeded by fundamental physical limits. Here we have shown that QDs have the potential to overcome the main deficiency of SPDC sources. Our device design can be used to boost the extraction efficiency of telecom QDs for fiber-based long-haul quantum communication. The broadband antenna is readily applicable to various optical-active materials. The efficient generation of entangled photon pairs demonstrated here provides a timely solution for the rapid development of quantum photonic technologies. Because of its brightness, this source is of particular interest for quantum communication protocols, quantum metrology or quantum imaging. Therefore, our result is an important step for the realization of a solid-state quantum information processing platform and could enable a new boost for fundamental research in quantum optics and quantum engineering.

## Methods

### Quantum dot growth

Samples were grown by solid source molecular beam epitaxy on GaAs (001) surface. After the growth of a GaAs buffer layer, 235 nm AlGaAs were deposited on a 50 nm AlAs sacrificial layer. During the deposition, the As_2_ cracker cell was kept at 650 degree. For nanohole fabrication, Al was deposited under low arsenic pressure ( < 10^−8^ mbar), forming droplets on the surface at 630 degree. These droplets etch into the substrate via As dissolution from the underlying GaAs substrate into the Al droplet, driven by concentration gradients at the interface. Then, the nanoholes were filled with 2 nm GaAs and subsequently overgrown by 142 nm AlGaAs to obtain the isolated QDs with three-dimensional carrier confinement.

### Device fabrication

The GaP semi-sphere lens has a radius of 1 mm and was fabricated from bulk material by mechanical polishing. We fabricate circular QD-containing membranes (with radius of 35 μm) by releasing them from the GaAs substrate following the silver layer deposition. We spin-coat the lens with PMMA at the speed of 7500 rpm and then picked up one membrane and placed it on the lens bottom. In the last step, the device is coated with alumina anti-reflection layer by atomic layer deposition.

### Two photon pumping

A pulsed Ti:Sa laser with 76 MHz repetition rate was used, which generated pulses with a duration of 3 ps. As the mode-locked laser shows broad emission, it is narrowed down spectrally. This is done by a home-built pulse shaping setup: The pulsed laser is dispersed by a 1200 l mm^−1^ grating to spread the laser photons of different energy spatially. A fiber is used to pick up desired photons. At the output port of the fiber, the laser power is measured as several milli-watt. The shaped laser pulse is then sent to QDs. In the collection beam path, we insert a notch filter (85–90% transmission when notching dip is 1 nm away) to filter out the scattering laser. Angle of notch filter is tuned carefully until the laser is maximally suppressed. Residual laser background noise is minimized by performing the experiment in a fiber-based closed-cycle cryostat system with a confocal setup where the fiber coupling is optimized for QDs emission.

### Data availability

The data that support the findings of this study are available from the corresponding author upon reasonable request.

## Electronic supplementary material

Supplementary Information
