# Supplementary material for: Highly-efficient extraction of entangled photons from quantum dots using a broadband optical antenna
Source: Nat Commun. 2018 Jul 31;9:2994. doi: 10.1038/s41467-018-05456-2 (PMC6068148; doi:10.1038/s41467-018-05456-2)
Supplement: Supplementary file 1 — Supplementary Information [file 41467_2018_5456_MOESM1_ESM.pdf]

Supplementary information-Highly-efficient extraction of entangled photons from quantum dots using a broadband optical antenna

Chen *et al.*

Supplementary Note1 - Quantum dot sample fabrication and its structure

The quantum dots (QDs) presented in this work are fabricated by solid source molecular beam epitaxy. In-situ droplet etching is utilized to create self-assembled nanoholes with ultrahigh in plane symmetry. These nanoholes are filled subsequently with GaAs to form the GaAs/AlGaAs QD. Supplementary Figure 1a shows a sketch of the processes involved in the QD formation. And Supplementary Figure 1b shows an AFM measurement and line scan in [110] and [1-10] direction, for a typical nanohole etched by local droplet etching. The cone-shaped hole has a dominantly circular base with a slight rhombic influence. The rhombic appearance might be an artifact of the shape of the AFM measurement tip. However, also measurements with conic tips still show a tendency to this shape. The average depth of the nanoholes is 12.2 nm, and the opening angle of the cone is around 66.5 degree. A ring of a height around 1-2 nm surrounds the dot, consisting of optically inactive AlAs (crystalized remains of the Al droplet). Due to negligible intermixing, the nanohole shape should reflect the QD shape. The final structure of the sample is summarized in Supplementary Table 1.

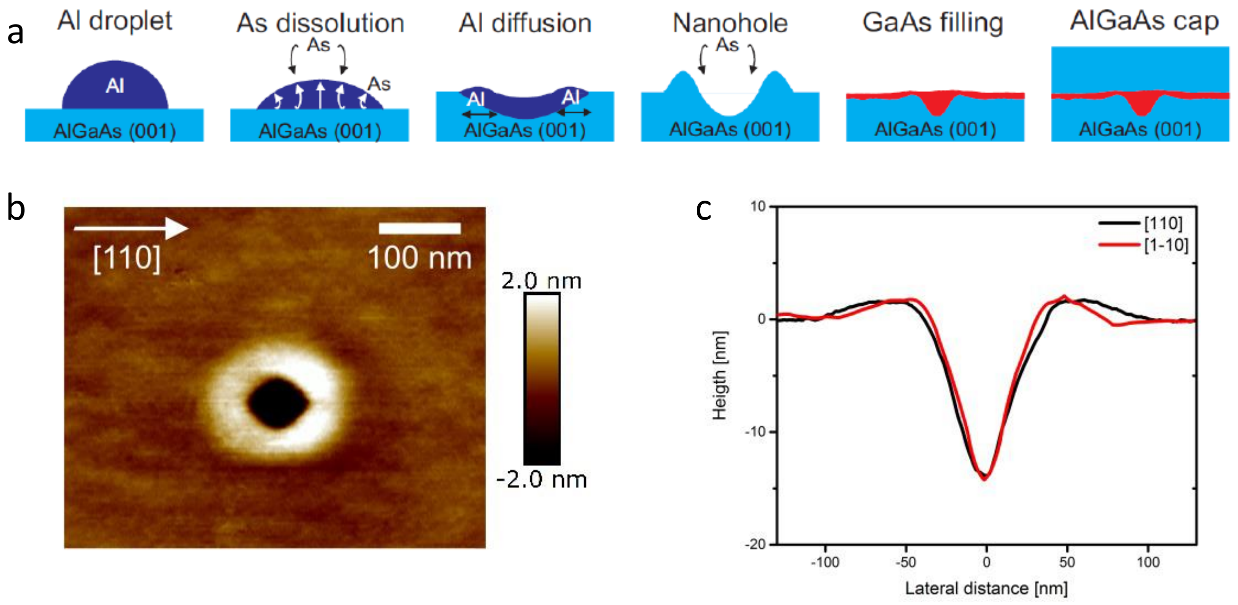

Supplementary Figure 1. Fabrication and characterization of a QD sample. (a) The fabrication process of a QD sample. (b) An AFM image of a droplet etched hole. The line scan across the hole show symmetric shape along both [110] and [1-10] directions (c).

Supplementary Table 1. Sample structure. A 50nm AlAs layer is grown as sacrificial layer. The QD layer is sandwiched in a bottom barrier layer (AlGaAs, 217nm) and a top barrier layer (AlGaAs, 160nm).

**Function**	**Material**	**Thickness**
**Buffer**	GaAs	200 nm
**Sacrificial layer**	AlAs	50 nm
**Bottom barrier**	AlGaAs	217 nm
**QD**	GaAs	2 nm
**Top barrier**	AlGaAs	160 nm

Supplementary Note 2 - Device fabrication

The fabrication of the antenna device starts from the preparation of the QD-containing membranes. The whole process is described in the following. Firstly, photolithography was used to define the size of membranes. Then 100nm silver layer was deposited on the sample by using e-beam sputtering. After the lift-off, the sample was etched in a mixture of wet chemical solvent consisting of sulfuric acid, hydrogen peroxide, and deionized water (ratio = 1:8:200). The etching process lasts around 4 min until the sacrificial layer is exposed. Then diluted HF (25%) was used to selectively etch away the sacrificial layer (AlAs). The membranes are now detached from the substrate and transferrable. The above processes are sketched in Supplementary Figure 2a. And in Supplementary Figure 2b, we show a picture of these detached membranes. One membrane is displaced a bit from its original position, verifying that the etching steps are successful.

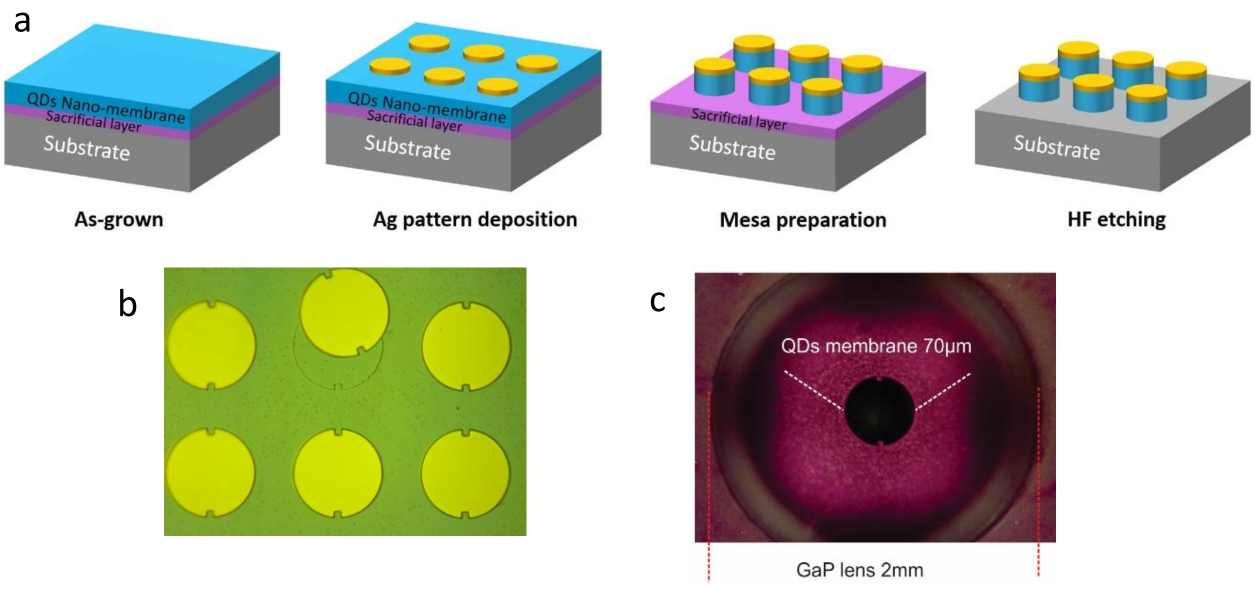

Supplementary Figure 2. Device fabrication. (a) The fabrication flow of QDs containing membranes. (b) An optical image QDs membranes after wet chemical etching. These membranes are already detached from the substrate. (c) An optical Image of an antenna device with QDs containing membrane in the center of a GaP semi-sphere lens.

Then we rinse the sample with water and let it dry. To transfer these membranes to a soft substrate, we place another substrate coated with photoresist (as glue) on top of the sample with slight pressure. After detaching, the QD-membranes get transferred to the substrate with the assist of photoresist. In the next step, we flush the QD-membranes off the substrate by acetone solvent on a cleanroom paper. Meanwhile we spin coat the GaP lens with PMMA at the speed of 7500 rpm. A 100nm thick PMMA layer is thus coated. We place the lens on top of a QD-membrane laying on cleanroom paper. By slight adjusting the position of the lens, we make sure one membrane sits in the center and then press the lens a little bit. The QD-membrane is picked-up and attached to the lens bottom. The microscope image of the final device is shown in Figure 2c. The black round disk in the center is the QDs containing membrane. It has a diameter of 70μm. The out ring is the edge of GaP lens. The image of the membrane is amplified by the lens.

Supplementary Note 3 - Numerical simulations

The calculation of the extraction efficiency of our device was performed in the framework of a finite element method by using a software package CAvity Modelling Framework, 'CAMFR' for short. Since the device has a rotational symmetry, a reduced 2D model structure was created. The QD emission is modeled as a horizontal dipole point source. The SIL is modelled as an infinite large medium. The total radiation is the sum of TE mode and TM mode radiations. The parameters used in the simulation are listed in table2.

Supplementary Table 2. Refractive indexes used in simulation

	AlGaAs	PMMA	silver	GaP
**Refractive index**	3.5	1.6	0.034+i*5.421	3.4

The coupling of photons from AlGaAs matrix to GaP lens depends sensitively on PMMA layer. We simulate the extraction efficiency against the radiation angle with different PMMA layer thicknesses. A 100nm PMMA layer is chosen in our device design. Simulations of the radiation field of the bulk substrate and antenna are presented in Supplementary Figure 3. Most of the photons are confined and dissipate within the substrate.

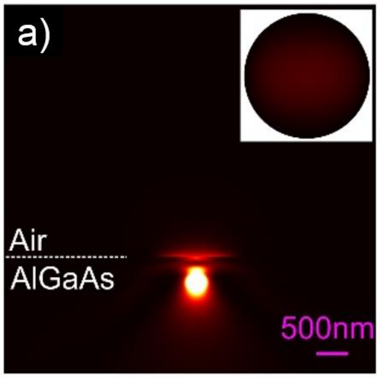

Supplementary Figure 3. Field distribution of QD emission in bulk AlGaAs substrate. Inset shows the upper far field distribution.

In order to extract both X and XX photons with high efficiency, the optical antenna needs to be operated at a broad spectral range. Theoretically, we calculate the extraction efficiency of photons at various wavelengths. The result is shown in Supplementary Figure 4. The extraction efficiency stays high over broadband, which confirms the broadband nature of our antenna.

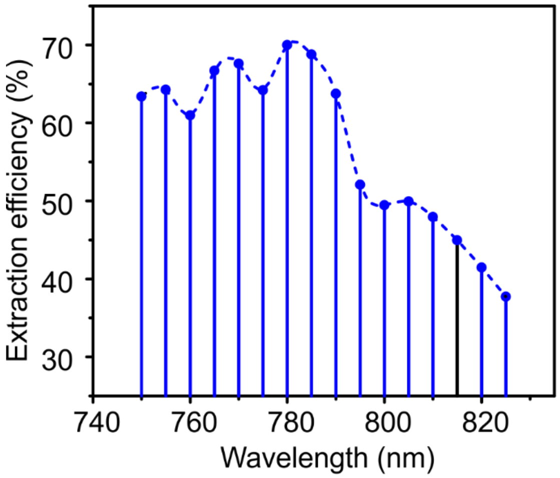

Supplementary Figure 4. Numerical calculation on the extraction efficiency for a broad wavelength range.

Supplementary Note 4 - Source brightness and Setup efficiency

A typical emission spectrum of a QD in the broadband antenna together with, for comparison, the spectrum of another QD in the bare substrate is shown in Supplementary Figure 5. An enhancement of a factor of more than 100x is clearly observed.

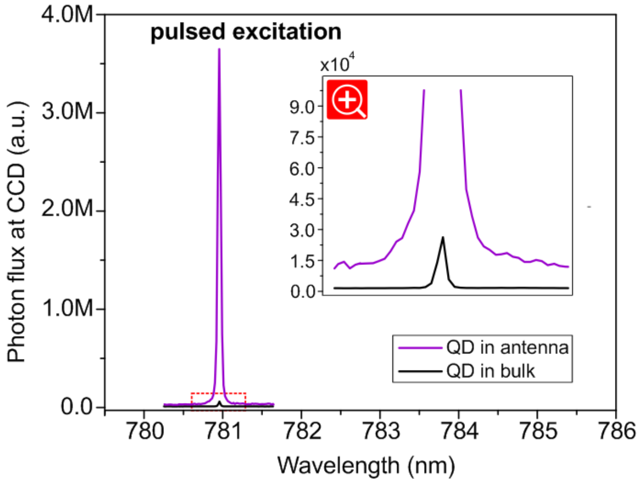

Supplementary Figure 5. Characterization of the antenna under 76MHz pulsed excitation. A typical PL spectrum with (purple) and without (black) the antenna. An enhancement of more than 100x is achieved. Inset zooms in on part of the spectrum.

A free-space helium flow cryostat depicted in Supplementary Figure 6a is used to quantify the extraction efficiency. The extraction efficiency is calibrated via mimicking QDs emission with laser. In a free space setup, the losses of every optical components are well calibrated. And the objective could collect all the photons even if the beam profiles of QDs emission and reflected laser slightly differs. Thus, the free space setup allows us to evaluate the extraction efficiency more precisely. The calibration method is similar with that in reference[1].

We first record the saturated count rate of the sample on APD under pulsed excitation which is 3.3MHz $\pm$ 0.1MHz. A silver mirror is then placed under the objective. The reflection ratio of this mirror is 97%. Then we tune the Ti:Sapphire laser to 780nm, the same wavelength as that of the QD emission, and focus it on the mirror. We increase the laser power, measure it right in front of the APD and also under the objective (see Supplementary Figure 6a) with an optical power meter. The laser intensities at these two locations are P1 = 2.8μw $\pm$ 0.2μw and P2 = 20 μw $\pm$ 0.5μw. Given the transmission of cryostat window (94%), the transmission from the antenna to the front of APD is expressed $T = (P1 \times 94\%)/(P2 \times 97\%)$ =13.6% $\pm$ 0.45%. The APDs we use are from Perkin-Elmer (SPCM-AQR-WX). The detection efficiency at 780 nm is 59% $\pm$ 2%. Since the recorded count rate is so high, we have to take the linearity correction factor of the APD into consideration. According to the manual, the actual photon rate could be calculated using the following equation:

$$\mathrm{ACTUALCOUNTRATE} = \frac{(OUTPUT\ ModuleCountRate \times CORRECTIONFACTOR@the\ Module\ CountRate) - DARK\ COUNT\ Module}{PHOTON\ DETECTION\ EFFICIENCY\ Module}$$

The Correction Factor follows this equation:

$$Correction\ Factor = \frac{1}{1-(t_d \times C_R)}$$

Where t_d is Module Dead Time and $C_R$is the Output Count Rate. With the provision of $t_d = 50ns$, the Correction Factor @3.3MHz is 1.2 and the actual count rate in front of the APD is 6.71MHz$\pm$0.3MHz. The extraction efficiency of the antenna η is therefore calculated as

$\frac{(ACTUALCOUNTRATE/T)}{LASER\ REPETITION\ RATE} = 65\% \pm 4\%$. The absolute detection efficiency of the whole optical setup is $\frac{OUTPUT\ ModuleCountRate}{PHOTON\ RATE\ AT\ FIRST\ LENS} \cong 6.7\%$.

To effectively populate XX state, two photon resonant excitation needs to be implemented. In such an excitation scheme, the laser lies very close with signal in wavelength. We need the fiber system to extinguish the laser. The setup is sketched in Supplementary Figure 6b. And all the relevant measurements are done under such an excitation scheme in this system. We also notice that for resonant excitation of QDs, it is a common method to characterize the device in a free space setup and measure other relevant data with a fiber based setup[2].

Assuming that X state can be excited (at saturation) with 100% fidelity under pulsed above-band excitation, the XX state population fidelity under two photon pumping scheme can be written as

$$\eta_{\mathrm{XX}} = \frac{R_{\mathrm{tpp}}}{R_{\mathrm{ab}}}.$$

Where R_{ab} is the count rate for above band excitation and R_{tpp} the count rate for two photon pumping. The XX state population fidelity is 88%$\pm 2\%$. Therefore the entangled photon pair rate $p = \eta_{\mathrm{XX}} \times \eta^2 = 0.372 \pm 0.002$. This pair rate makes our source a) 3 times brighter than the brightest QD source, b) ~5 times brighter than today's brightest SPDC sources, c) more than 1000 times brighter than other broadband sources. What's more, the high entanglement fidelity and easy device fabrication we demonstrated here are also big advantages.

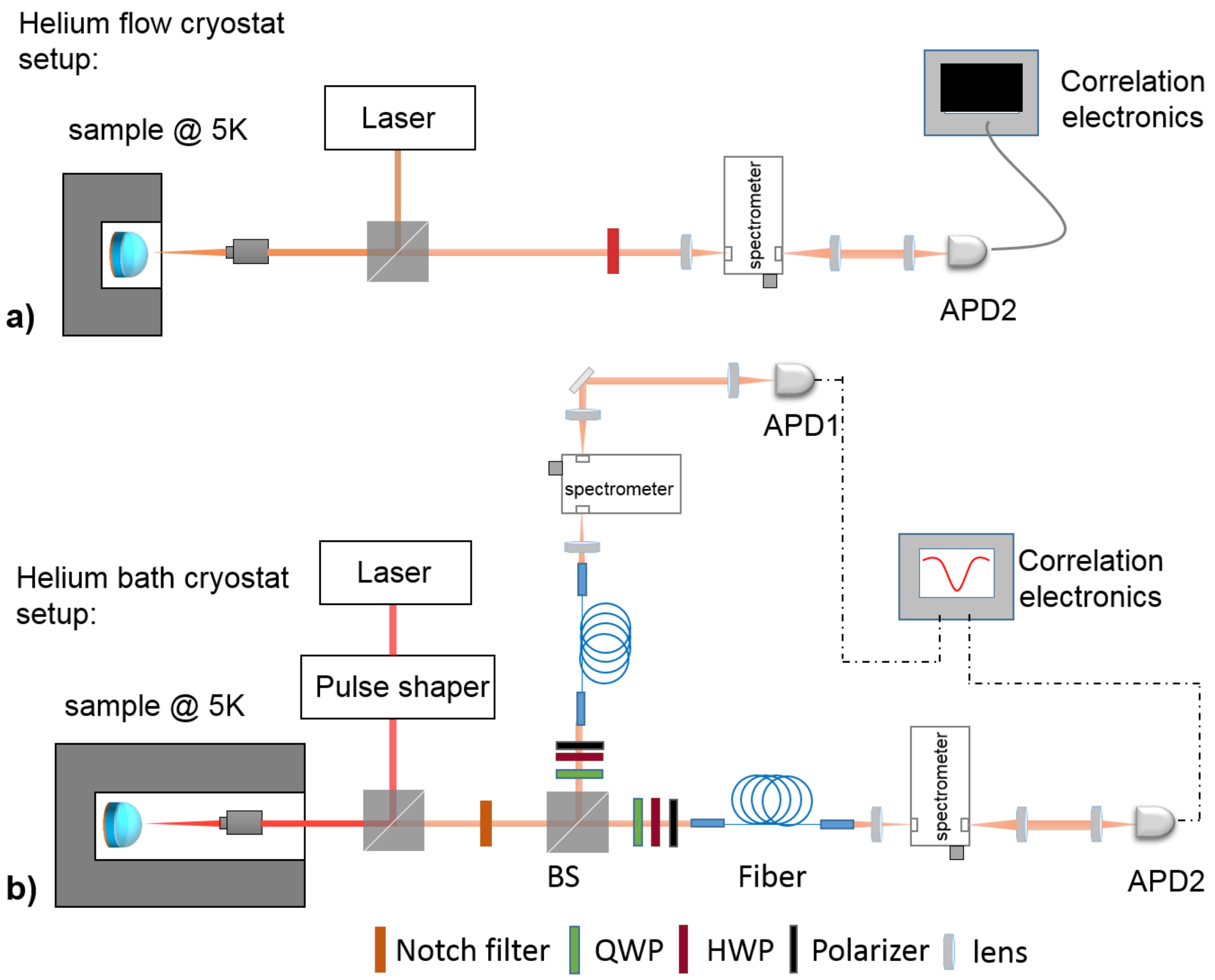

Supplementary Figure 6. Photoluminescence setup. (a)Optical setup with a helium flow cryostat system. (b)Optical setup with a closed-cycle helium bath system. Compared with system (a), more optical components are inserted into beam pass to measure entanglement fidelity.

Supplementary Note 5 - Resonant two-photon pumping with continuous wave laser.

Thanks to the highly efficient excitation/collection, we are be able to resonantly excite the XX state with a continuous wave (*c.w.)* laser. The wavelength of *c.w.* laser is tuned in between of X and XX transitions. The recorded PL is shown in Supplementary Figure 7. Considering the short lifetime of X and XX, the photon pair rate can in principle go to the very exciting Gigahertz range with increased laser power.

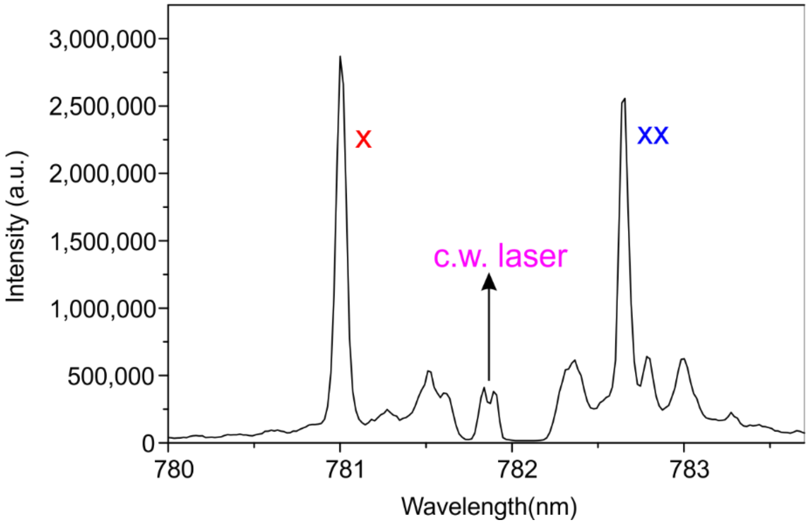

Supplementary Figure 7. Resonant excitation of XX state with a continuous wave laser. Strong peaks of X and XX emission are observed. Laser was filtered out by narrow band notch filters.

Supplementary Note 6 - Lifetime of X and XX

The lifetime is measured with a fast APD. Single exponential decay was used to fit the XX decay data, while consecutive exponential decay mode was used to fit X decay data as X is populated by the XX decay in the cascade. After devolution with system response function, the lifetime for X and XX are 195 ps and 103 ps, respectively.

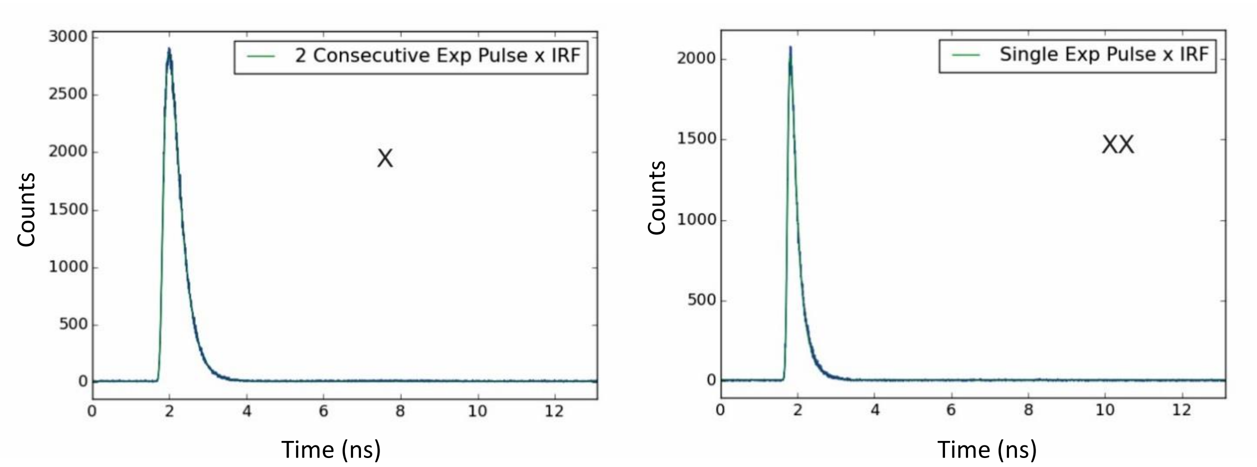

Supplementary Figure 8. Photoluminescence lifetime. (Left)Lifetime for X. (Right) Lifetime for XX. Blue line is the experimental date and green line the fitting curve.

Supplementary Note 7 - Entanglement tomography

16 sets of cross correlation were measured to evaluate the tomography. The results are shown in Supplementary Figure 9. Method to exact the entanglement fidelity are described in Reference[3]. The calculated entanglement fidelity is 89%.

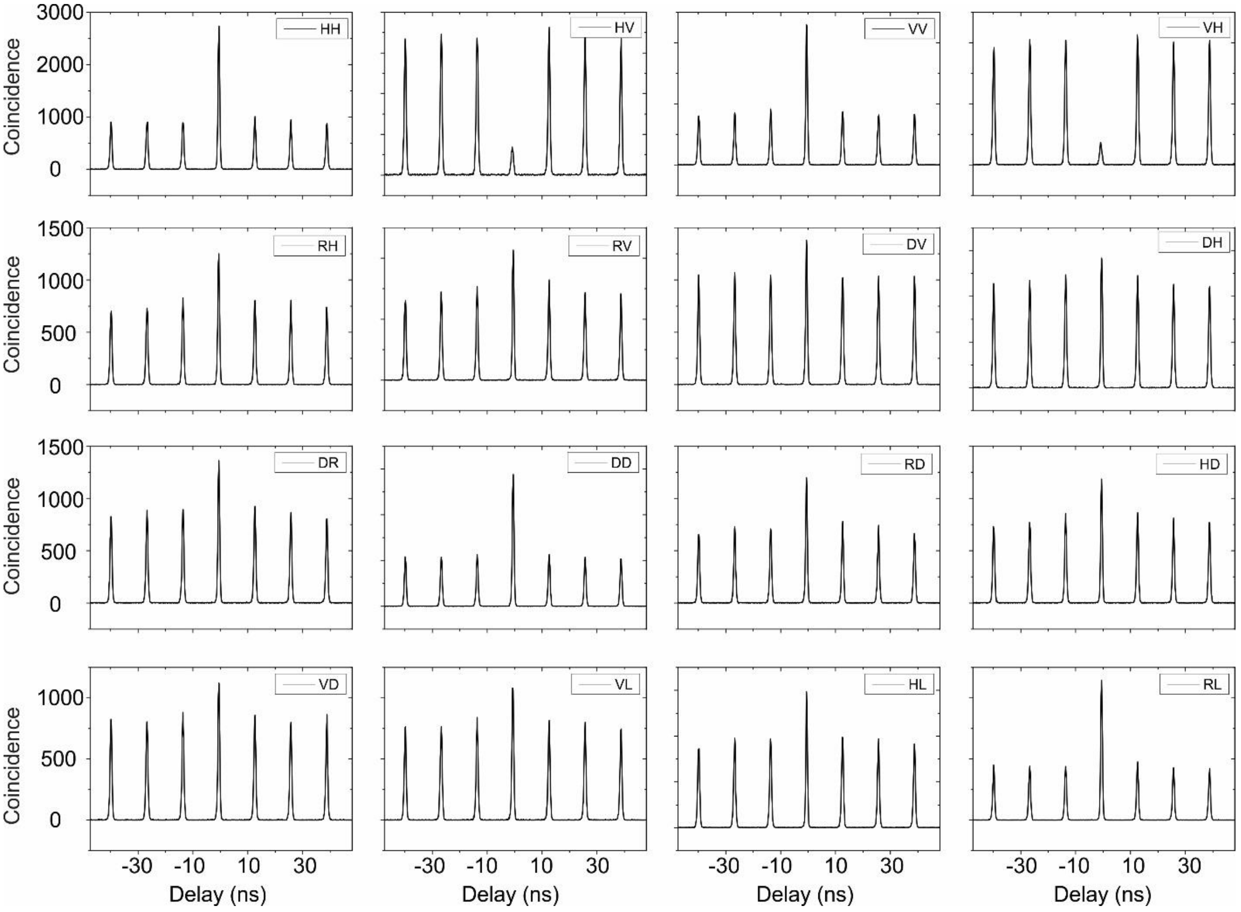

Supplementary Figure 9. Entanglement tomography.16 sets of cross-correlation are measured. The calculated entanglement fidelity is 89%.

Supplementary References

1. Claudon, J. et al. A highly efficient single-photon source based on a quantum dot in a photonic nanowire. *Nat Photon* **4**, 174-177 (2010).
2. SomaschiN et al. Near-optimal single-photon sources in the solid state. *Nat Photon* **10**, 340-345 (2016).
3. James, D.F.V., Kwiat, P.G., Munro, W.J. & White, A.G. Measurement of qubits. *Physical Review A* **64**, 052312 (2001).
